# Supplementary material for: COZOID: contact zone identifier for visual analysis of protein-protein interactions
Source: BMC Bioinformatics. 2018 Apr 6;19:125. doi: 10.1186/s12859-018-2113-6 (PMC5889581; doi:10.1186/s12859-018-2113-6)
Supplement: Supplementary file 2 — Software build. Executable binary file of the software tool. (ZIP 118,784 kb) [file 12859_2018_2113_MOESM2_ESM.zip › cozoid/platform/docs/testng-6.5.1-javadoc.zip!/org/testng/IRetryAnalyzer.html]

IRetryAnalyzer (TestNG)


---


|  |  |  |
| --- | --- | --- |
| **Overview** \| **Package** \| **Class** \| **Tree** \| **Deprecated** \| **Index** \| **Help** | |  |
| **PREV CLASS**   **NEXT CLASS** | **FRAMES**    **NO FRAMES**     **All Classes** |  |
| SUMMARY: NESTED \| FIELD \| CONSTR \| METHOD | DETAIL: FIELD \| CONSTR \| METHOD |  |


---


## org.testng Interface IRetryAnalyzer

---

``` public interface IRetryAnalyzer ```

Interface to implement to be able to have a chance to retry a failed test.

**Author:**
:   tocman@gmail.com (Jeremie Lenfant-Engelmann)

---

| **Method Summary** | |
| --- | --- |
| `boolean` | `retry(ITestResult result)`             Returns true if the test method has to be retried, false otherwise. |

**Method Detail**

### retry

```
boolean retry(ITestResult result)
```

:   Returns true if the test method has to be retried, false otherwise.

    :   **Parameters:**: `result` - The result of the test method that just ran. **Returns:**: true if the test method has to be retried, false otherwise.


---


|  |  |  |
| --- | --- | --- |
| **Overview** \| **Package** \| **Class** \| **Tree** \| **Deprecated** \| **Index** \| **Help** | |  |
| **PREV CLASS**   **NEXT CLASS** | **FRAMES**    **NO FRAMES**     **All Classes** |  |
| SUMMARY: NESTED \| FIELD \| CONSTR \| METHOD | DETAIL: FIELD \| CONSTR \| METHOD |  |


---
